# Remedy for Night Sweats in Phthisis

**Published:** 1880-04

**Authors:** 


### REMEDY FOR NIGHT SWEATS IN PHTHISIS.

KÖHNBORN (*Berliner Klinische Wochenschrift*, Jan. 5th. 1880,) states, that in two cases, in which he had tried all other remedies in vain, he met with the most surprising success in treating the profuse night sweating of phthisis, by means of the powder which is employed by the Military Medical Department of the War Minister, for the treatment of sweating of the feet. This is composed of salicylic acid three, starch ten, and talc eighty-seven parts. The entire body is to be powdered with this in the evening, the patient protecting the mouth and nose by means of a handkerchief, lest the irritation from the salicylic acid might induce coughing. If the skin is very dry, the powder may be made to adhere to it by first rubbing it with fat bacon or spirits and tannin.—*Medical and Surgical Reporter.*

